# Spontaneous necrosis of a single digit: watershed necrosis

**DOI:** 10.1080/23320885.2021.1874385

**Published:** 2021-02-01

**Authors:** Alain J. Azzi, Gabriel Bouhadana, Fanyi Meng, Peter G. Davison

**Affiliations:** a Division of Plastic and Reconstructive Surgery, McGill University, Montreal, Canada; b Faculty of Medicine, McGill University, Montreal, Canada

**Keywords:** Necrosis, fingers, case reports, arteriogram, palmar arch

## Abstract

Anatomical variations in the superficial and deep palmar arches are common, but rarely lead to digital necrosis. We report the case of necrosis of the third digit caused by a ‘watershed’ effect in the context of atherosclerotic disease and rare congenital variations of the superficial and deep palmar arches.

## Introduction

Classically, the radial and ulnar arteries enter the hand and anastomose across the palm to form the superficial and deep palmar arches. Normally, the main supply of the deep palmar arch (DPA) is the radial artery, whereas the superficial palmar arch (SPA) is ulnar-dominant. The DPA usually branches to form the princeps pollicis and the radial digital artery of the second digit. The SPA gives off the ulnar digital artery of the fifth digit and the common palmar digital arteries in the second, third and fourth web spaces. Vascular redundancy and interconnections render the blood supply to the hand and digits robust. Examples include interconnections between the deep and superficial arches, between the palmar metacarpal arteries and the digital arteries, between the dorsal metacarpal arteries and the digital arteries, etc. Anatomical variations are common and do not usually pose a risk to digit blood supply due to this network of interconnections. We present a case of spontaneous ‘watershed’ necrosis of a single digit caused by arterial disease, a pathology that would have otherwise been benign in patients with normal anatomy. To our knowledge, this mechanism of digit necrosis has yet to be reported in the literature.

## Case report

**Figure 1.**
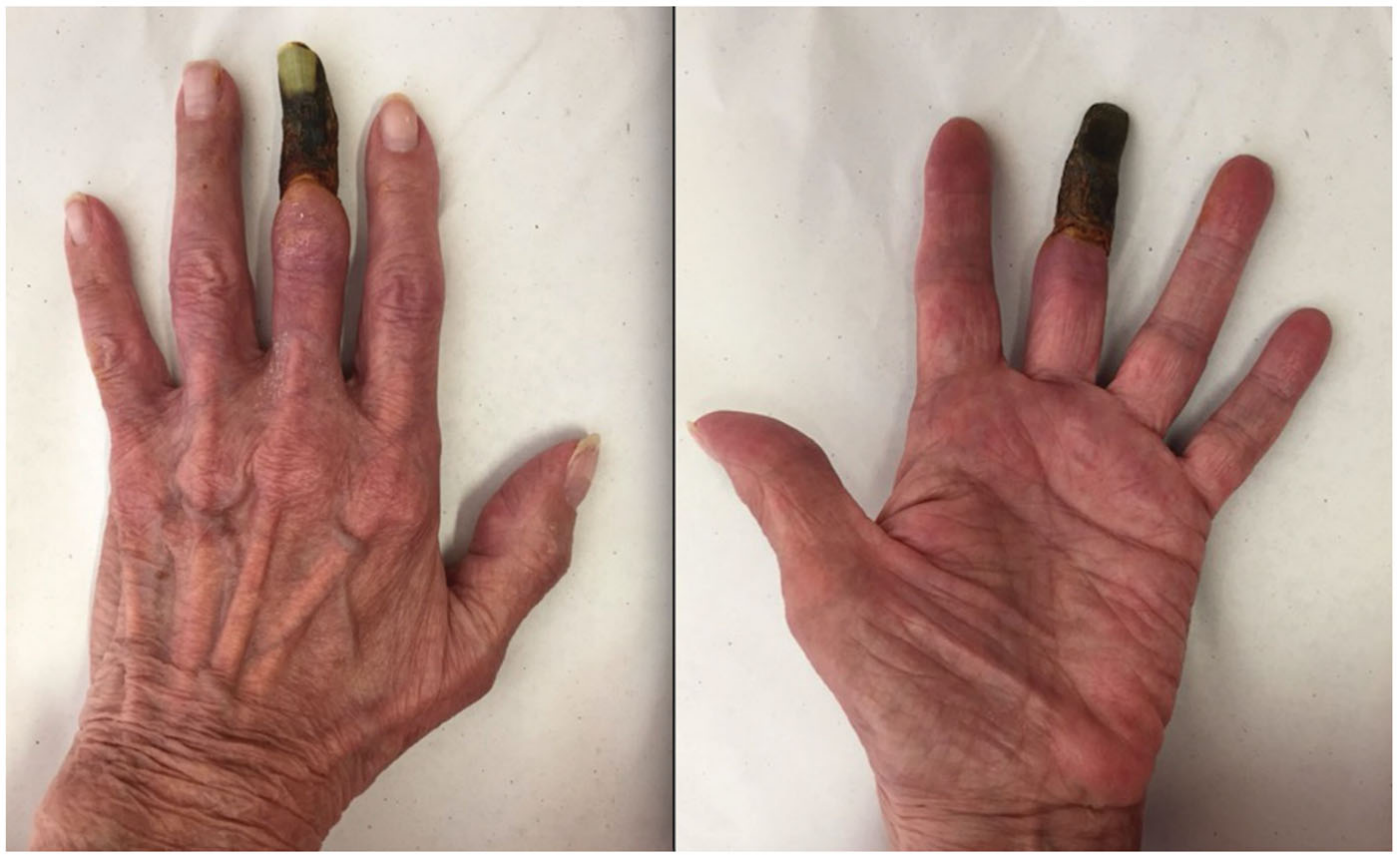
A dorsal and palmar view of the patient’s hand at presentation, demonstrating necrosis of the middle finger distal to the middle phalanx.

A 75-year-old patient presented to our hand clinic for spontaneous necrosis of the left third digit, distal to the middle phalanx (Figure 1). The patient is an ex-smoker (30 pack-years) and is known for hypertension, palindromic rheumatism and pernicious anemia. She described progressive worsening of the digit over a period of two weeks. Discoloration began after she was holding an electronic tablet with her finger wrapped around the top for a prolonged period of time. It is unclear whether the prolonged pressure exerted on the finger was related to the insult, but the patient describes it as the initiating event.

**Figure 2.**
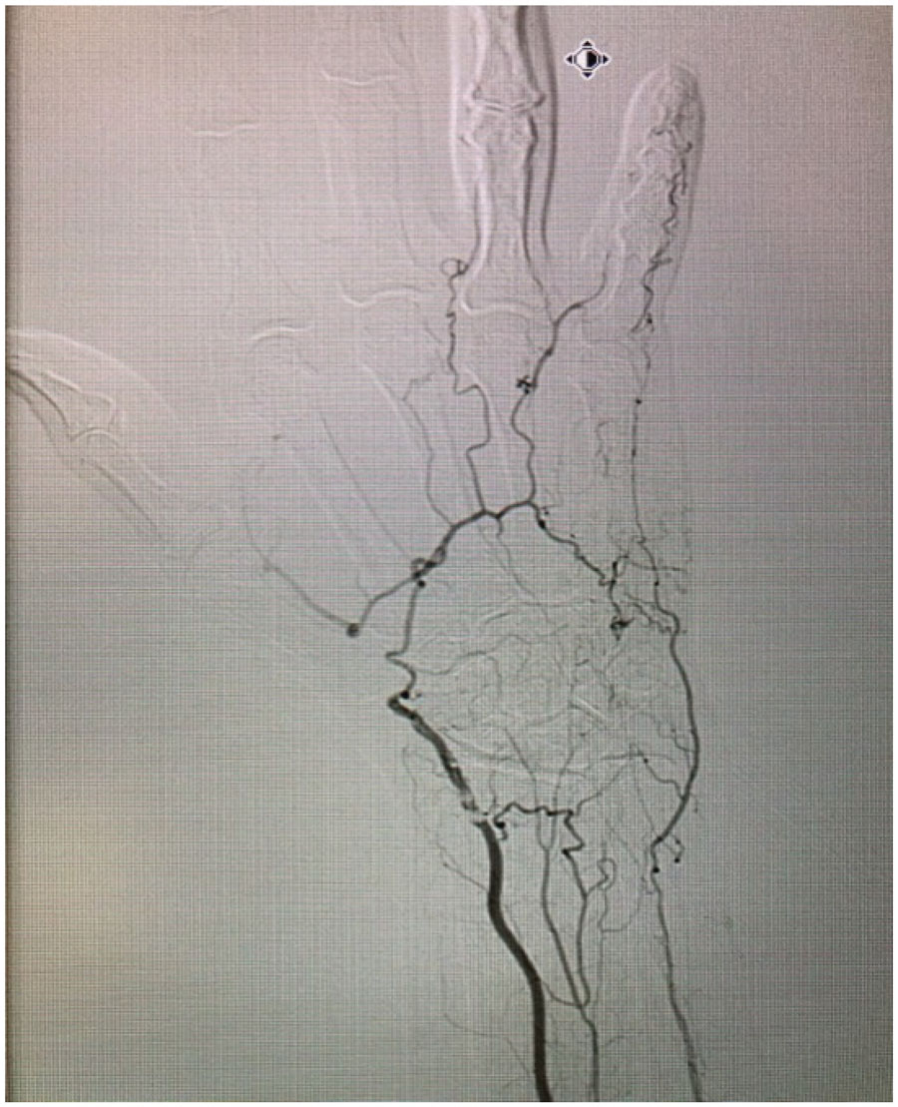
Left hand arteriogram, demonstrating 80% stenosis of the radial artery at the level of the wrist, suggestive of an atheromatous plaque, absence of the DPA, an incomplete SPA at the level of the fourth and fifth digits (collaterals were seen bridging the incomplete superficial arch).

On examination, necrosis was noted, demarcated at the mid-middle phalanx of the third digit (Figure 1). No other skin lesions were noted and there were no signs of infection. The digit demonstrated decreased range of motion and absent sensory findings. The patient denied any symptoms of claudication, a history of prolonged cold exposure, or sensitivity to cold. Laboratory tests revealed normal liver and renal function and were not suggestive of systemic etiologies for the necrosis. The patient had no signs of an underlying inflammatory process nor any anemia, thrombocytosis or elevated C-Reactive Protein count. Albeit that the patient was positive for Rheumatoid Factor and anti-cyclic citrullinated peptide (anti-CCP) antibodies, this comes to no surprise as her rheumatism was a longstanding disease, with a remote history of flare ups that were well-controlled with trials of prednisone. Although a progression of the patients’ palindromic rheumatism into a rheumatoid vasculitis is possible, it is highly unlikely due to absence of clinical evidence of rheumatological decompensation other than digital necrosis and absence of any laboratory findings (the patient was negative for any antinuclear, anti-Smith or anti-RNP antibodies). An x-ray of the hand was unremarkable. A left upper extremity arteriogram was ordered which showed several significant findings: 80% stenosis of the radial artery at the level of the wrist, suggestive of an atheromatous plaque, absence of the DPA, an incomplete SPA at the level of the fourth and fifth digits (collaterals were seen bridging the incomplete superficial arch) and no sign of vascularity distal to the base of the middle phalanx of the third digit (Figure 2 and Supplemental Online Material 1).

Despite several investigations by rheumatology ruling out a hypercoagulable state or other systemic causes, hematology was consulted for antiplatelet therapy. The patient was eventually booked for a thrombectomy +/- bypass of the radial artery with a cephalic vein graft and an amputation of the necrotic third digit. Intra-operatively, the radial artery lumen was found to be occluded by the plaque. A segment of 4 cm was resected and bridged with a cephalic vein graft. Strong pulsatile flow was confirmed before closure. Unfortunately, the patient had a repeat angiogram on the tenth post-operative day showing complete occlusion of the radial artery, suggesting thrombosis at the anastomosis. The patient was subsequently referred to our vascular surgery colleagues who ordered upper extremity pressure studies, which revealed Digital Brachial Indices ranging from 0.34 to 0.51 in the left hand, and individual finger pressures ranging from 62 to 92 mm Hg. This is in contrast to the right index finger which demonstrated a pressure of 168 mm Hg and a Digital Brachial Index of 0.93. The above findings further support arterial disease as the cause of necrosis, which was particularly problematic due to the patient’s aberrant anatomy and lack of normal anastomoses and redundancies.

## Discussion

Case reports of spontaneous digit necrosis are mainly related to vascular pathologies, including Takayasu’s arteritis, Buerger’s disease, and either cryoglobulinemia or Raynaud’s phenomenon when preceded by exposure to cold, to name a few [1–4]. Buerger’s disease would classically present in a younger patient, with a history of smoking and of joint complaints, or other end organ ischemia, which were not present in our patient. Moreover, angiography did not reveal ‘corkscrew vessels’. Takayasu’s was considered less likely as our patient had no limb claudication pain or constitutional symptoms, while the absence of any dermatologic signs such as papules or ulcers made cryoglobulinemia or calcinosis cutis less likely. Moreover, most case reports in the literature describe necrosis of multiple digits, while the patient in the present case report developed spontaneous necrosis of a single digit. This was due to common arterial disease, a pathology that would have otherwise been benign in patients with normal anatomy. With the input of our rheumatology colleagues, no systemic cause for necrosis was identified in our patient.

We present a case of spontaneous digit necrosis due to an incomplete SPA paired with a suspected absence of the DPA. The most likely cause for the potential absence of the DPA is congenital in nature, given the complete absence of its visualization. While we acknowledge the possibility of the DPA not having been visualized due to complete atherosclerotic occlusion or hypoperfusion, we maintain that these are less likely given the complete absence of visualization of any part of the DPA or of any contrast distally. However, irrespective of the cause of absence of the DPA, the patient’s anatomy is nonetheless unique and precarious in nature. In addition to the reduction of the radial artery contribution to the SPA due to an atherosclerotic occlusion, the ulnar blood supply of the SPA was also reduced due to the incomplete nature of the arch between the fifth and the fourth digits, bridged only by collaterals. The third digit was therefore in a ‘watershed’ area of the SPA, positioned in the middle of the arch and receiving marginal blood supply from either direction. With this in mind, it is worthy to consider that the resulting necrosis of the third digit is thus highly suggestive of the absence of the DPA. A complete DPA would have provided potential compensation for decreased SPA flow, a phenomenon that could have occurred in a patient with normal anatomy due to interconnections between the DPA and SPA. Moreover, the palmar metacarpal artery, normally originating from the DPA, would have potentially supplied the third digit due to its interconnections with the common digital artery. To our knowledge, this is the first case report to demonstrate spontaneous necrosis of a digit due to a unique anatomical variation of the SPA, in combination with absence of the DPA and advanced vascular disease.

Although a classical pattern exists, it is important to note that anatomical variations of the DPA and SPA are common, primarily due to their complex embryological origins. Up until day 56, the palmar arches are still under embryological development, maturing from proximal to distal, starting off as a capillary plexus that eventually becomes well-defined vessels [5]. First defined and classified by Jaschtschinski in 1897, an incomplete arch is one where there is an absence of anastomosis between the vessels constituting the arch [6]. Many attempts at classifying the collateral circulation of the hand have been made. Most notably, these include Gellman et al. who have subclassified the variations of the SPA into five subgroups for complete arches and two for incomplete arches, while subclassifying the variations of the DPA into three main groups [7]. Coleman and Anson have also proposed a classification for the SPA, which includes five subgroups for complete arches and four for incomplete arches [8]. None demonstrated an absence of direct anastomosis of the SPA at the level of the fourth and fifth digits. On this basis, we were unable to categorize the patient presented in this case report in any of the classification systems present in the literature. The prevalence of complete and incomplete palmar arches has been reported in the literature, but show significant inter-researcher variations. The prevalence of an incomplete SPA, which is suspected in our patient, was reported to range all the way from 3% to as high as 68%. In a meta-analysis regrouping of all of these studies, Zarzecki et al. reported the prevalence of an incomplete SPA to be at 18.7% [9]. An incomplete DPA is much less common and a complete absence of the DPA, such as in our patient, is extremely rare [10]. The literature is devoid of data to provide an accurate estimate of the prevalence of an incomplete DPA.

While our patient received a cephalic vein graft and a digital amputation at the middle phalanx, treatment options in similar situations can vary depending on the cause and chronicity of the ischemia. Medical management is reserved for chronic disease with no evidence of ischemia/ulceration and is targeted at mitigating sympathetic hyperactivity and vasospasm [11]. When the etiology is occlusive in nature, revascularization is the treatment of choice (e.g. thrombectomy, reconstruction of the radial/ulnar artery with vein grafts or reconstruction of the palmar arches using the dorsal venous arch, to name a few). A Digital Brachial Index of less than 0.7, inadequate collateral circulation and segmental occlusion with distal ‘run off’ all serve as indications for revascularization [12–14]. If irreversible damage has occurred and necrosis has developed, amputation is necessary. Allowing the digit to auto-amputate is a reasonable option in the absence of infection. However, if a ray amputation is indicated (third or fourth digit necrosis proximal to the proximal interphalangeal joint), it should be performed early to expedite recovery and return to work.

In summary, the vasculature of the hand is designed to compensate and resist digit hypoperfusion from atherosclerotic disease. The palmar arches and their interconnections usually provide digits with adequate blood supply, even in the context of extensive atherosclerotic disease. From a practical point of view, the uniqueness of our patient’s anatomy resulted in an uncommon and previously undescribed mechanism of digit necrosis, analogous to watershed hypoperfusion that occurs in the brain or intestines. From a clinical point of view, this uncommon presentation highlights the intricacy of the hand blood supply and the impact of anatomical variations.

## Supplementary Material

Supplemental Material
